# Drug Interactions at the Human Placenta: What is the Evidence?

**DOI:** 10.3389/fphar.2012.00126

**Published:** 2012-07-09

**Authors:** Miriam Rubinchik-Stern, Sara Eyal

**Affiliations:** ^1^School of Pharmacy, Institute of Drug Research, The Hebrew UniversityJerusalem, Israel

**Keywords:** placenta, pregnancy, maternal-fetal pharmacology, drug interactions, P-glycoprotein, breast cancer resistance protein, organic cation transporters

## Abstract

Pregnant women (and their fetuses) are treated with a significant number of prescription and non-prescription medications. Interactions among those drugs may affect their efficacy and toxicity in both mother and fetus. Whereas interactions that result in altered drug concentrations in maternal plasma are detectable, those involving modulation of placental transfer mechanisms are rarely reflected by altered drug concentrations in maternal plasma. Therefore, they are often overlooked. Placental-mediated interactions are possible because the placenta is not only a passive diffusional barrier, but also expresses a variety of influx and efflux transporters and drug-metabolizing enzymes. Current data on placental-mediated drug interactions are limited. In rodents, pharmacological or genetic manipulations of placental transporters significantly affect fetal drug exposure. In contrast, studies in human placentae suggest that the magnitude of such interactions is modest in most cases. Nevertheless, under certain circumstances, such interactions may be of clinical significance. This review describes currently known mechanisms of placental-mediated drug interactions and the potential implications of such interactions in humans. Better understanding of those mechanisms is important for minimizing fetal toxicity from drugs while improving their efficacy when directed to treat the fetus.

## Introduction

Pregnant women (and their fetuses) are exposed to a significant number of prescription and non-prescription medications. In North America, women use an average of 2.3 prescription medications during pregnancy, but many consume four drugs or more (Mitchell et al., 2011). Often, these drugs are taken throughout pregnancy, for the treatment of chronic diseases, such as hypertension, asthma, and epilepsy. As the pregnancy rates among women 40 years of age and older have been steadily increasing (Ventura et al., 2008), the mean number of drugs used by a pregnant woman is expected to increase in future years. Those polytherapeutic regimens may result in drug–drug interactions (DDIs) that affect the mother, the fetus, or both. DDIs that involve the fetus can result from changes in plasma concentrations (unbound or total) of at least one of the interacting drugs, changes in drug’s disposition or effects within the fetus, or a combination of the two. A third source for altered fetal effects of drugs resides in the interfaces between plasma and the fetus, namely the placenta. As with several other distributional DDIs, placental-mediated DDIs may be rarely reflected by altered drug concentrations in maternal plasma, especially when only a small fraction of the total amount of drug in the body distributes into the fetus (Endres et al., 2006).

By modulating placental function, a drug can potentially affect the distribution of another drug into the fetus, its removal from the fetus, or both. These changes may be of clinical relevance, because the outcomes of *in utero* exposure to medications may vary from benign to structural and behavioral teratogenicity and termination of pregnancy, and are often unknown.

## The Placental Barrier

The feto-placental-maternal circulation is established around the 10th week of pregnancy. The placenta then becomes a permeability boundary which regulates the exchange of nutrients and metabolites between mother and fetus (van der Aa et al., 1998). Within the placenta, the barrier between maternal and fetal circulations consists of trophoblast cells and to some extent the endothelium of fetal capillaries. As the pregnancy proceeds, the cytotrophoblast layer partially disappears. Consequently, the trans-placental diffusion distance decreases, increasing placental permeability. Transport across the placenta then becomes greatly regulated by the two membranes of the syncytiotrophoblast, the syncytial microvillous membrane at the maternal side, and the basal membrane at the fetal side (van der Aa et al., 1998).

**Figure 1:**
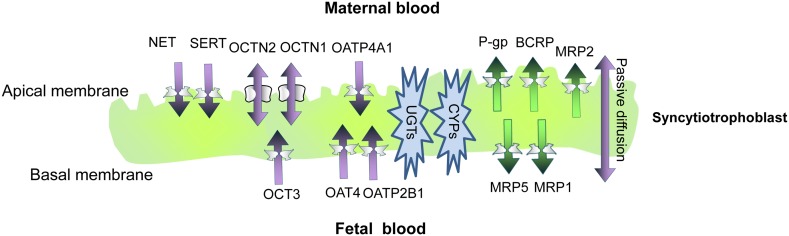
**A schematic representation of the syncytioblast**. Free drugs and their metabolites usually cross the placenta by passive diffusion, transporter-mediate transfer, or both. Within the syncytium, drugs can undergo phase I and phase II metabolism. P-gp, P-glycoprotein; BCRP, breast cancer resistance protein; MRP, multidrug resistance-associated protein; OATP, organic anion-associated polypeptide; OAT, organic anion transporter; OCT, organic cation transporter; OCTN, organic cation/carnitine transporters; SERT, serotonin transporter; NET, norepinephrine transporter; ENT, equilibrative nucleoside transporter; CYP, cytochrome P450; UGT, uridine diphosphate glucuronosyltransferases.

The placental barrier can limit the delivery of drugs targeted to treat the fetus, or given to prevent maternal-to-fetal disease transmission, such as in the case of anti-HIV-protease inhibitors (Endres et al., 2006). At the same time, the barrier may protect the fetus against the effect of noxious compounds. An example is the chemotherapeutic agent doxorubicin, which has been safely used in pregnancy (Cardonick and Iacobucci, 2004). Yet, most drugs that enter the maternal circulation can cross the placenta to some extent (Unadkat et al., 2004). Passive diffusion is the predominant route by which drugs cross the placenta, and applies to molecules of less than 600 Da, which are hydrophobic and non-ionized. However, an important role for membrane transporters in placental drug transfer is being increasingly recognized (Figure 1). Phagocytosis and pinocytosis have not so far been shown to significantly contribute to placental drug transfer (Ni and Mao, 2011). Of note, drugs which diffuse rapidly across the placenta may display blood flow-limited transfer kinetics, and their delivery to the fetus may be affected by medications which alter placental blood flow. In addition, developmental changes in the placenta that occur during pregnancy, such as reduced trophoblast thickness, increased surface area and enhanced placental blood flow, may alter the rate of trans-placental diffusion over pregnancy (Unadkat et al., 2004).

### Placental drug-metabolizing enzymes

The placenta expresses a variety of xenobiotic-metabolizing enzymes from the earlier stages of pregnancy. However, compared to the liver, placental drug metabolism seems to be relatively minor and does not significantly limit the extent of drug delivery to the fetus. On the other hand, placental enzymes can catalyze the formation of reactive metabolites that might be fetotoxic (Pasanen and Pelkonen, 1994). Drugs which have been shown to undergo significant placental metabolism include azidothymidine, dexamethasone, and prednisolone (van der Aa et al., 1998). The anticonvulsant oxcarbazepine (but not carbamazepine) is also metabolized to some extent by the human placenta (Pienimäki et al., 1997; Myllynen et al., 1998).

Placental enzymes catalyze phase I (drug oxidation, reduction, and hydrolysis) and Phase II (conjugation) reactions. Among phase I enzymes proteins which have been identified in the placenta are cytochrome P450 (CYP) 1A1, 2E1, 3A4, 3A5, 3A7, 4B1, and 19 (Syme et al., 2004; Myllynen et al., 2009). At the mRNA level, the major isoforms expressed are placental aromatase (CYP19) and CYP11A (Nishimura et al., 2003). Placental CYP19 is involved in the metabolism of several drugs, including the antidiabetic agent glyburide (Zharikova et al., 2009) and the opioids buprenorphine (Deshmukh et al., 2003) and methadone (Nanovskaya et al., 2004). Furthermore, methadone may act as a potent inhibitor of CYP19 *in vivo*, thus potentially interfering with steroid hormone metabolism and drug detoxification (Deshmukh et al., 2003; Lu et al., 2010). In mothers treated with the glucocorticoid betamethasone, placental CYP19 mRNA content and aromatase activity were significantly lower than in controls (Paakki et al., 2000). Whether these changes affect placental drug metabolism and fetal exposure to drugs and their metabolites is currently unknown.

The most established placental phase II enzymes with regard to xenobiotic metabolism are uridine diphosphate glucuronosyltransferases (UGTs). For example, in the trans-placental perfusion model, 17% of the antipsychotic drug olanzapine was conjugated to the *N*-glucuronide (Schenker et al., 1999). Other placental phase II enzymes include glutathione *S*-transferase, epoxide hydrolase, *N*-acetyltransferase, and sulfotransferases.

The type and the amount of drug-metabolizing enzymes change over gestation. In general, more CYP isozymes are expressed in the first trimester, when the fetus is more susceptible to the effects of teratogens, than at term (Hakkola et al., 1996). In contrast, UGTs are present at the placental metabolic barrier throughout pregnancy. Placental xenobiotic-metabolizing activities are affected by a variety of maternal and environmental factors, including drug abuse, smoking, alcohol consumption, polluted air, and contaminated food (Stejskalova and Pavek, 2011). For example, UGT and CYP1A activities are significantly elevated in placentae of mothers who smoke and are greatest in women who both smoke and consume alcohol (Collier et al., 2002b). In rats, treatment with the anticonvulsant phenobarbital induced placental CYP3A1 (Ejiri et al., 2005a) but did not affect the expression of phase II enzymes (Ejiri et al., 2005b). To our knowledge, no significant DDIs which involve placental phase II enzymes have been reported so far. However, the presence of UGTs at the placental metabolic barrier throughout gestation implies a major role for them in xenobiotic detoxification (Collier et al., 2002a). Their modulation is therefore expected to affect the amount of substrate drugs transferred to the fetus.

### Placental transporters

Placental transporters mediate the uptake of hormones and nutrients from maternal blood into the fetus and the substrate removal back to maternal blood. Transporters therefore may contribute to the barrier which protects the fetus from toxins and teratogens. Furthermore, placental transporters take part in the homeostasis of placental tissue itself (Ganapathy, 2011; Ni and Mao, 2011). Uptake transporters facilitate substrate influx into the syncytium and fetal capillary cells, whereas efflux transporters export their substrates from cells, although some transporters can exhibit bidirectional substrate transport. For many drugs, the net transfer across the placenta may be determined by interplay between several transport systems which can operate in the same direction or opposite directions. Furthermore, the impact of a transporter on substrate transfer across the placenta would not be substantial if the substrate undergoes extensive trans-placental diffusion.

Two major superfamilies of transporters are present in the placenta, adenosine triphosphate binding cassette (ABC), and solute carrier (SLC) transporters (Vähäkangas and Myllynen, 2009; Prouillac and Lecoeur, 2010). This section will focus on transporters which have been reported or are likely to be involved in DDIs at the placenta.

#### Transporters of the adenosine triphosphate binding cassette superfamily

Adenosine triphosphate binding cassette transporters are primary active transporters which function to remove substrates from cells. Most of the recognized placental ABC transporters are located at the maternal-facing (apical) brush border membrane of the syncytiotrophoblast (Figure 1). There, they play a major role in decreasing the fetal exposure to drugs and other xenobiotics (Ni and Mao, 2011). The most abundant ABC transporters on the apical membrane of syncytiotrophoblasts, and likely the most clinically relevant ones, are P-glycoprotein (P-gp) and the breast cancer resistance protein (BCRP; Vähäkangas and Myllynen, 2009). The multidrug resistance proteins (MRPs) 1–3 and 5 have also been identified in the human placenta (Ni and Mao, 2011).

##### P-glycoprotein

P-gp (MDR1), which is expressed at the apical membrane of syncytiotrophoblasts, is encoded by the *ABCB1* gene (Ceckova-Novotna et al., 2006). In rodents, two corresponding multidrug resistance proteins are encoded by the mdr1a and mdr1b genes (Schinkel et al., 1997). P-gp substrates are generally hydrophobic molecules, of which many are cationic. Substrate drugs include HIV-protease inhibitors, immunosuppressive drugs, and antibiotics. P-gp activity can be inhibited by therapeutic compounds, including those administered to pregnant women such as the immunosuppressant cyclosporine A (CsA) and the cardiovascular agent verapamil (Unadkat et al., 2004).

The role of P-gp in protecting the fetus from potential teratogens was first demonstrated in CF-1 mice, that are naturally deficient of mdr1a. Dams were exposed to an isomer of the pesticide avermectin, a P-gp substrate known to produce cleft palate in mice. Fetuses homozygous for P-gp deficiency were 100% susceptible to cleft palate, whereas in heterozygote fetuses the incidence of this malformation was 30%. The homozygous wild-type fetuses were insensitive to avermectin at the doses tested (Lankas et al., 1998). Further evidence for the protective role of P-gp was provided by Smit et al. (1999). Mdr1a/b^+/−^ heterozygous females were mated with heterozygous males. Intravenous administration of ^3^H-digoxin, ^14^C-saquinavir, and paclitaxel to the dams resulted in 2.4-, 7-, and 16-fold greater concentrations, respectively, of the administered drug in Mdr1a/b^−/−^ fetuses than in their wild-type siblings. In the same study, oral administration of the P-gp inhibitors valspodar or elacridar to pregnant Mdr1a/b^+/+^ mice produced similar increases in drug distribution to that observed in Mdr1a/b^−/−^ fetuses, indicating that placental P-gp can be completely inhibited in mice by pharmacological means. In perfused rat placentae, coadministration of CsA with quinidine or chlorpromazine resulted in 1.7- and 1.9-fold increases, respectively, in CsA concentration in the fetal compartment (Pávek et al., 2001).

**Figure 2:**
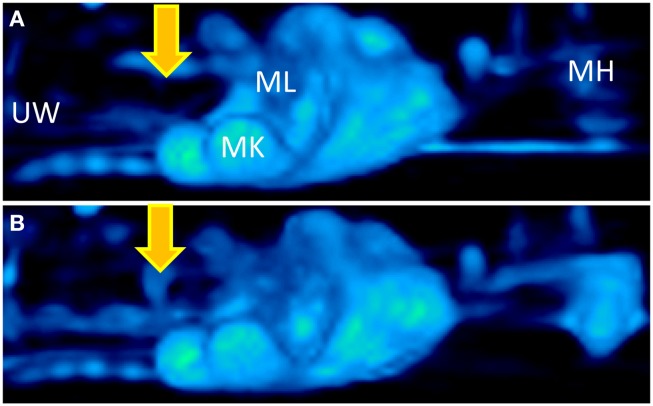
**Three dimensional projections of PET images of ^11^C-verapamil radioactivity in pregnant *Macaca nemestrina* before (A) and during (B) administration of cyclosporine A (CsA; 12 mg/kg/h)**. Fetal liver was the reporter of ^11^C-radioactivity that crossed the placental barrier. Because of P-gp inhibition, CsA significantly increased the distribution of ^11^C-radioactivity into fetal liver. Thus, the fetal liver is seen in the figure only in B (indicated by yellow arrows). MH, maternal head; MK, maternal kidneys; ML, maternal liver; UW, uterine wall. Based on studies conducted by Eyal et al. (2009).

More recently, positron imaging tomography (PET) was used to study the contribution of placental P-gp to fetal protection *in vivo* in a more representative model, the non-human primate. ^11^C-verapamil and CsA were used as the model P-gp substrate and inhibitor, respectively. In this study, the significance of placental P-gp was demonstrated by the 2.2-fold increase in fetal liver radioactivity after CsA administration during late pregnancy (Eyal et al., 2009). Of note, maternal plasma concentrations of CsA were greater compared to those achieved in clinic. Additionally, in this study and in those conducted in mice, the impact of transporter inhibition at the placenta was consistently less, compared to that observed at the blood-brain barrier (Figure 2; based on studies conducted by Eyal et al., 2009).

In the dually perfused human placenta model, P-gp inhibition increased the maternal-to-fetal transfer of the HIV-protease inhibitors saquinavir (Mölsä et al., 2005), indinavir, and lopinavir (Ceccaldi et al., 2009; Table 1). Consistent with those observations, the cord-to maternal concentration ratios of indinavir and saquinavir are extremely low, and that of lopinavir is lesser than one (Hutson et al., 2011). Further studies demonstrated a role for P-gp in the maternal-to-fetal transfer of the chemotherapeutic agent paclitaxel, the opioid methadone (Nanovskaya et al., 2005), and the beta-blocker talinolol (May et al., 2008; Table 1). Moreover, in human placental membrane vesicles, methadone, buprenorphine, and morphine competitively inhibited paclitaxel transport (Hemauer et al., 2009). In contrast, in perfused term placentae, maternal-to-fetal transfer of digoxin was not affected by quinidine or verapamil (Holcberg et al., 2003).

**Table 1:** **Effect of transporter inhibition on drug transfer across perfused term human placentae**.

Transporter	Drug (concentration)	Inhibitor (concentration)	Fold change in maternal-to-fetal transfer in inhibitor-treated vs. control placentae(P)^a^	Fold change in fetal-to- maternal transfer in inhibitor-treated vs. control placentae (P)^a^	Reference
P-gp	Saquinavir (6.7 μg/mL)	PSC833 (2.4 μg/mL)	3.5 (<0.01)	0.7 (>0.05)	Mölsä et al. (2005)
		GF120918 (0.6 μg/mL)	3.0 (<0.01)	ND
	Indinavir (7.6 μg/mL)	PSC833 (1.2 μg/mL)	1.5 (<0.05)	ND	Sudhakaran et al. (2008)
		Ritonavir (0.22 μg/mL)	1.1 (>0.05)	ND
	Lopinavir (6 μg/mL)	CsA (12.0 μg/mL)	10.3 (<0.05)	ND	Ceccaldi et al. (2009)
	Talinolol (0.29 μg/mL)	Verapamil^b^ (13.6 μg/mL)	1.2 (<0.05)	ND	May et al. (2008)
		PSC833 (2.2 μg/mL)	1.0 (>0.05)	ND
	Paclitaxel (0.085 μg/mL)	GF120918 (0.6 μg/mL)	1.7 (<0.01)	ND	Nanovskaya et al. (2005)
	Methadone (0.2 μg/mL)	GF120918 (0.6 μg/mL)	1.3 (<0.01)	ND
MRPs	Talinolol (0.29 μg/mL)	Probenecid^c^ (2.9 μg/mL)	1.2 (<0.05)	ND	May et al. (2008)
	Saquinavir (6.7 μg/mL)	MK-571 (26.9 μg/mL)	ND	0.9 (>0.05)	Rahi et al. (2009)
		Probenecid^c^ (14.3 μg/mL)	ND	0.8 (>0.05)
OCTs	Metformin (2.0 μg/mL)	Cimetidine (100 μg/mL)	1.3 (>0.05)	1.3 (>0.05)	Tertti et al. (2010)

*The methodology for calculation of maternal-to-fetal and fetal-to-maternal transfer varied across studies. ^a^Values are normalized to changes in the transfer of markers for passive diffusion. ^b^Also an MRP1 and OATPs inhibitor. ^c^Also an OATPs inhibitor. ND, not determined*.

P-gp has been detected in syncytiotrophoblast cells throughout gestation. However, expression of P-gp mRNA and protein decreases with gestational age (Ni and Mao, 2011). The exact factors which regulate P-gp expression have not been identified, although a role has been suggested for estrogen, progesterone, glucocorticoids, and various cytokines (Ni and Mao, 2011). Indeed, in animal models, the synthetic glucocorticoids betamethasone (Kalabis et al., 2009) and dexamethasone (Petropoulos et al., 2010) affected placental P-gp expression, although changes in P-gp protein expression did not translate into altered placental transfer of ^3^H-digoxin (Petropoulos et al., 2010). Interestingly, P-gp expression in term placentae was greater in HIV-1-infected women receiving pharmacotherapy than in uninfected controls (Camus et al., 2006).

##### The breast cancer resistance protein

Similar to P-gp, placental BCRP has been localized to the brush border membrane of syncytiotrophoblast cells (Figure 1). In addition, BCRP is expressed on the luminal (fetal-facing) surface of placental capillaries (Ni and Mao, 2011). BCRP substrates include many drugs used during pregnancy, such as nitrofurantoin and glyburide (Ni and Mao, 2011). Some BCRP substrates are also substrates of P-gp and MRPs (Ni and Mao, 2011). Importantly, HIV-protease inhibitors are not transported by BCRP. Instead, drugs of this class, such as ritonavir, saquinavir, and nelfinavir, are effective BCRP inhibitors (Gupta et al., 2004). Other BCRP inhibitors which may be used by pregnant women include CsA (Gupta et al., 2006) and omeprazole (Breedveld et al., 2004). Unlike P-gp, the data about the expression levels of BCRP in the placenta throughout gestation are not consistent (Ni and Mao, 2011).

In P-gp deficient pregnant mice (to avoid confounding drug transport by P-gp) orally treated with elacridar, fetal penetration of topotecan increased twofold, compared to wild-type controls (Jonker et al., 2000). Similarly, studies with Bcrp1^−/−^ pregnant mice treated with glyburide (Zhou et al., 2008) and nitrofurantoin (Zhang et al., 2007) demonstrated 2.3- and 5.0-fold increase, respectively, in the fetal/maternal drug concentration ratio compared to wild-type fetuses. In the perfused human placenta model, the BCRP inhibitor nicardipine increased 1.8-fold the fetal-to-maternal concentration ratio of glyburide (Pollex et al., 2008).

##### Multidrug resistance-associated proteins

Multidrug resistance proteins are predominantly organic anion transporters but in addition transport neutral organic compounds. The substrate and inhibitor selectivity of individual MRPs may partially overlap with that of other MRPs, P-gp, BCRP, and organic anion transporters (Hagenbuch and Meier, 2004; Ni and Mao, 2011). Unlike P-gp and BCRP, knowledge on the functional significance of placental MRPs is relatively poor, and data related to potential MRPs-mediated placenta DDIs are currently available for MRPs 1 and 2 only.

Multidrug resistance protein 1 is predominantly expressed on fetal blood vessel endothelia and the basal membrane of the term human syncytiotrophoblast (Figure 1). Some expression has also been detected at the apical layer of syncytiotrophoblasts (St-Pierre et al., 2000). In BeWo trophoblast cells, CsA, a P-gp, and MRP1 inhibitor, increased the net apical-to-basolateral transfer of saquinavir, a substrate for both P-gp and MRPs (Park and Sinko, 2005). However, in a later study, the MRP1 inhibitor probenecid did not affect the trans-placental transfer of saquinavir (Rahi et al., 2009; Table 1).

Multidrug resistance protein 2 is expressed at the apical human syncytiotrophoblast membrane (St-Pierre et al., 2000; Figure 1). A functional role of MRP2 in the placental barrier for xenobiotics was demonstrated by May and coworkers. Slight but statistically significant increases in maternal-fetal permeability of talinolol were demonstrated in the presence of the MRP2 inhibitor, probenecid, and the non-specific inhibitor, verapamil, but not by the P-gp inhibitor PSC833 (May et al., 2008; Table 1).

#### Transporters of the solute carrier superfamily

Solute carrier transporters known to be expressed in the placenta and involved in trans-placental drug transfer include organic anion transporting polypeptides, organic anion transporters, organic cation transporters (OCTs), monoamine transporters, and equilibrative nucleoside transporters. Among these, currently available data point mostly to potential DDIs which involve OCTs or monoamine transporters.

##### Organic cation transporters

Organic cation transporters include the potential-sensitive OCTs and the proton gradient-driven OCTNs. Among the human OCTs, only OCT3 (SLC22A3) is expressed in the human placenta, at the basal membrane of trophoblast cells (St-Pierre et al., 2002; Figure 1). In addition, both OCTN1 and OCTN2 are expressed at the maternal-facing membrane of syncytiotrophoblasts, where they likely facilitate substrate transfer from the maternal to the fetal side (Ganapathy and Prasad, 2005; Figure 1). OCTN 1 and 2 substrates include verapamil and quinidine (Ciarimboli, 2011). It has been suggested that metformin transport across the placenta is mediated by OCTs (Kovo et al., 2008). However, a later study showed lack of significant effect of the OCT inhibitor cimetidine on the placental transfer of metformin (Tertti et al., 2010; Table 1).

##### Monoamine transporters

The serotonin transporter (SERT) and the norepinephrine transporter (NET), which are responsible for the neuronal uptake of these monoamines, are expressed on the maternal-facing membrane of syncytiotrophoblasts (Figure 1; Ganapathy, 2011). Amphetamines, but not cocaine, are substrates for the two transporters. A variety of antidepressants are established inhibitors of monoamine transporters and their impact on placental SERT and NET has yet to be clarified (Ganapathy, 2011).

## Impact of Maternal Pharmacokinetics on Fetal Exposure to Drugs

Fetal exposure to medications depends not only on their transfer across the placenta but also on their pharmacokinetics in both mother and fetus. As pregnancy precedes, related physiological changes, including changes in organ blood flow, hepatic drug-metabolizing enzyme activity (Anderson, 2005), and renal elimination (Hebert et al., 2008; Eyal et al., 2010) may affect the overall amount of drugs and their metabolites that cross the placenta. Moreover, if placental transfer mechanisms are saturable, as in the case of metformin (Kovo et al., 2008), such changes over gestation could result in non-linear trans-placental transfer kinetics and alter the magnitude of placental-mediated DDIs. Finally, altered fetal exposure to medications may be caused by simultaneous interactions among drugs at multiple maternal and fetal organs (e.g., inhibition of drug-metabolizing enzymes or transporters in maternal tissues, the placenta, and potentially fetal tissues themselves).

## Conclusion

The majority of currently reported DDI at the placenta involve ABC efflux transporters, in particular P-gp. Based on studies in rodents, it has been assumed that such interactions can significantly affect fetal exposure to medications. Studies in isolated human placenta suggest that efflux transporters are indeed important in limiting drug delivery to the fetus. However, the magnitude of such interactions appears to be modest, and overall lesser compared to those observed at the blood-brain barrier. To our knowledge, the impact of such interactions in terms of fetal outcomes in humans has not been reported so far. Nevertheless, for substrates such as lopinavir, P-gp inhibition can significantly affect fetal exposure. Whether P-gp inhibition can enhance fetal toxicity from drugs with narrow therapeutic index (e.g., doxorubicin) is currently unknown. It is still not clear whether the substrate-inhibitor combinations studied so far represent other drug combinations, and whether the magnitude of interaction that occurs in term placentae is representative of various disease conditions and earlier gestational ages. For example, because P-gp expression decreases during pregnancy, the effect of its inhibition may be greater in early gestation, during fetal organogenesis. In addition, interactions may be mediated by yet unidentified transporters and other components of the placenta. It should be also kept in mind that drugs can potentially affect the delivery of hormones and nutrients to the fetus. Therefore, additional data are needed with other substrates and inhibitors for optimization of drug choice. Manipulation of placental transporters has been suggested as means to control fetal exposure to medications and enhance the efficacy of pharmacotherapy in the fetus (Ito, 2001; Syme et al., 2004). However, more studies are required before such interventions can be recommended. Future studies should address the potential sources for the different results obtained in humans vs. rodents and establish models for prediction of placental-mediated DDIs. Importantly, incorporating measurements of drug concentrations in maternal and cord plasma in longitudinal studies in pregnant women and their children may improve our understanding of the outcomes associated with polytherapy during pregnancy.

## Conflict of Interest Statement

The authors declare that the research was conducted in the absence of any commercial or financial relationships that could be construed as a potential conflict of interest.

## Abbreviations

ABC, adenosine triphosphate binding cassette; AUC, area under the concentration-time curve; BBB, blood-brain barrier; BCRP, breast cancer resistance protein; CsA, cyclosporine A; DDI, drug-drug interaction; MDR, multidrug resistance protein; MRP, multidrug resistance-associated protein; OATP, organic anion transporting polypeptide; OCT, organic cation transporter; OCTN, organic cation/carnitine transporter; P-gp, P-glycoprotein; SLC, solute carrier.
